# Citizen science reveals alarming update on the invasion of the Asian mantleslug *Meghimatium pictum* in Brazil

**DOI:** 10.1371/journal.pone.0330518

**Published:** 2025-09-02

**Authors:** Rafael M. Rosa, Daniel C. Cavallari, Marcel S. Miranda, Fernanda S. Silva, Rodrigo B. Salvador

**Affiliations:** 1 Center for Marine Biology, University of São Paulo, São Sebastião, Brazil; 2 Department of Zoology, Institute of Biosciences, University of São Paulo, São Paulo, Brazil; 3 Center for Biodiversity Documentation, Departament of Biology, Faculty of Philosophy, Sciences and Letters at Ribeirão Preto, University of São Paulo, Ribeirão Preto, Brazil; 4 Reef Biology Laboratory, Department of Biological Oceanography, Oceanographic Institute, University of São Paulo, São Paulo, Brazil; 5 Museum of Zoology, University of São Paulo, São Paulo, Brazil; 6 Zoology Unit, Finnish Museum of Natural History, University of Helsinki, Helsinki, Finland; Universidade Estadual de Feira de Santana, BRAZIL

## Abstract

The Asian mantleslug *Meghimatium pictum* is an exotic species introduced to Brazil in the late 1990s, but only formally reported in 2011. Since then, it has been deemed an agricultural pest and given the status of an invasive species; furthermore, it has been confirmed as an intermediate host for the nematode *Angiostrongylus costaricensis* in Brazil. Despite its potential for impacts, no additional studies on the status of its invasion have been conducted since the initial report. In this study, we used the citizen science platform iNaturalist to analyse the current distribution of *M. pictum* in Brazil, while also using genetic barcode data to understand the relationships between Brazilian and Asian populations and applying a species distribution model to investigate the suitable range for its distribution in Brazil. Our survey has recovered a total of 520 new records of this species in Brazil, confirming its spread to four additional states since its original report. Currently, *M. pictum* is recorded in the Distrito Federal and the states of Minas Gerais, Espírito Santo, Rio de Janeiro, São Paulo, Paraná, Santa Catarina, and Rio Grande do Sul. Our phylogenetic analysis suggests a close relationship between *M. pictum* populations in South America and those from Okinawa, Taiwan, and Guangzhou (mainland China), the latter being the most likely point of origin for the South American populations. Our species distribution model supports the idea that, in Brazil, the species is largely restricted to subtropical areas belonging to the Atlantic Forest ecoregion, while also showing suitable areas where the species has not been recorded yet and might become established in the near future. The implications of these findings are discussed, highlighting the recent surge in records and the usefulness of iNaturalist as a tool to monitor invasive species in the country.

## Introduction

The Asian mantleslug *Meghimatium pictum* (Stoliczka, 1873) is a herbivorous terrestrial slug native to China. It is typically found in humid environments and has been documented in a variety of habitats, including trees, hollow trunks, stones, human buildings, and waste disposal sites [1]. This species belongs to the family Philomycidae, which includes the Asian genus *Meghimatium* and the American genera *Phylomicus*, *Pallifera,* and *Megapallifera* [2]. The Philomycidae are distinguished from other land slugs by having a mantle covering their entire dorsal region, earning them the common name of mantleslugs [1,2].

Beyond its native range, *Meghimatium pictum* has been introduced in several countries worldwide, including Argentina and Brazil [1,3,4]. In Brazil, the earliest recorded occurrence of *M. pictum* dates back to 1998, with a specimen collected in the municipality of Curitiba, in the state of Paraná. Since then, the species has been repeatedly documented in the states of São Paulo, Paraná, Santa Catarina, and Rio Grande do Sul [1]. Despite the collection of several specimens since the late 1990s, the presence of *M. pictum* in Brazil was only formally recognised in 2011. Before that, published records incorrectly identified the specimens as belonging to related American species in the genera *Pallifera* and *Philomycus* [1,5–9].

This species has received little attention since its initial discovery in Brazil, which is especially worrying considering the significant time elapsed since its introduction. Previous studies have shown that *M. pictum* has already spread to several protected areas in southern Brazil [1], reported to cause agricultural losses in grape crops [10], and found to be an intermediate host for the parasitic nematode *Angiostrongylus costaricensis* Morera & Céspedes, 1971 [11,12]. Over a decade has passed since the last comprehensive review of the occurrence of *Meghimatium pictum* in Brazil [1], and the current extent of its invasion remains unknown.

In recent years, the citizen science platform iNaturalist has been consistently used as a powerful tool in assessing the distribution of species [13–16], including in the study of both native and exotic terrestrial gastropods in Brazil [17,18]. With this in mind, we analysed the records of *Meghimatium pictum* on iNaturalist to understand its current distribution in Brazil, used genetic barcode data to understand the relationships between Brazilian and Asian slug populations, and applied species distribution modelling to understand the suitable range for its distribution in Brazil.

## Materials and methods

The data used in this study were gathered from the iNaturalist platform (https://www.inaturalist.org/) on February 09, 2025, and analyzed following the methodology established in our previous studies [17,18]. Observations on the platform were filtered by taxon (*Meghimatium pictum*) and country (Brazil). Each record was then individually reviewed to ascertain the identity of the observed animals as *M. pictum*.

*Meghimatium pictum* can be readily distinguished from other slug species in Brazil, both native and exotic, based on the following external characters: an elongated cylindrical body with a rounded anterior margin and a pointed posterior margin; mantle covering the entire dorsal region; long head extending from beneath the anterior margin of the mantle; sole occupying the entire ventral body region; visible respiratory pore as a short slit near the anterior right margin of the mantle; background color of mantle yellowish to opaque beige, with two dark brown to black longitudinal lateral stripes, one often lighter medial stripe and scattered dark brown irregular spots or short lines surrounding the medial and lateral stripes; foot sole and head cream colored, with dark ocular tentacles [1]. Most or all of these characters are visible in photographs, making them useful for identification in iNaturalist observations. The only species also recorded in Brazil with a somewhat similar appearance to *M. pictum* are the related *Pallifera* species, but they can be easily distinguished based on their different colour patterns [1].

The list of *M. pictum* records in Brazil can be found in S1 File.

### DNA barcoding and phylogenetic analysis

Since the seminal report of Gomes et al. [1], further genetic barcode sequences of *M. pictum* from Asia and South America have become available. That prompted us to place the Brazilian populations into a phylogenetic context to try to understand their relationships with Asian populations. To that end, we used sequence data from previous studies deposited on GenBank (Table 1) and also sequenced one additional specimen (voucher CMRP 1117, deposited in the Coleção Malacológica de Ribeirão Preto, Faculdade de Filosofia, Ciências e Letras de Ribeirão Preto, University of São Paulo, Ribeirão Preto, Brazil). This specimen was collected in the littoral region of São Paulo state and likely represents one of the first established populations of *M. pictum* in Brazil, as São Paulo’s coast has the largest port area in Latin America, recognised as a prime port of entry for exotic species [e.g., 22,23].

**Table 1:** GenBank accession numbers of COI sequences used in the phylogenetic analysis, with data on the place of origin of the specimen sequenced and reference to the original publication.

Species	GenBank acc. nr.	Place of origin	Reference
*Meghimatium baoshanense* Tsai, Lu & Kao, 2011	FJ896668	Taiwan, Kaohsiung, Tengjhih	[19]
	FJ896672	Taiwan, Nantou, Sitou	[19]
*Meghimatium bilineatum* (Benson, 1842)	EF128225	Taiwan, Nantou, Jiji	[2]
	FJ896631	China, Zhejiang, Xiu Shan	[19]
	FJ896634	Japan, Okinawa, Southeast Botanical Garden	[19]
	LC754979	Vietnam, Lạng Sơn, Chi Lăng, Vân Thủy	[20]
	MN022745	Mauritius	[21]
*Meghimatium burchi* Tsai & Wu, 2008	EF105117	Taiwan, Nantou, Koan-Tau Mt.	[2]
	FJ896611	Taiwan, Taichung, Syueshankeng	[19]
*Meghimatium fruhstorferi* (Collinge, 1901)	EF128223	Taiwan, Taoyuan, Fusing, Dongyan Mt.	[2]
	FJ896646	Japan, Okinawa, Nago	[19]
	FJ896650	Japan, Fukuoka, Itoshima, Shima	[19]
	LC754786	Japan, Hokkaido, Shimamaki	[20]
*Meghimatium pictum* (Stoliczka, 1873)	EF128224	Taiwan, Taipei, Wulai, Taman	[2]
	FJ896651	Taiwan, Taichung, Wushikeng	[19]
	FJ896652	Taiwan, Taichung, Yousheng River	[19]
	FJ896653	Taiwan, Nantou, Lugu	[19]
	FJ896654	Taiwan, Pingtung, Wanluan	[19]
	FJ896655	Taiwan, Taitung, Litao	[19]
	FJ896656	Taiwan, Taitung, Lanyu	[19]
	FJ896657	Taiwan, Hualien, Ruisui	[19]
	FJ896658	Taiwan, Nantou, Meifeng farm	[19]
	FJ896659	Taiwan, Taipei, Rueifang, Jiufen	[19]
	FJ896660	Taiwan, Taipei, Wulai, Taman	[19]
	FJ896661	Taiwan, Kinmen, Kinhu	[19]
	FJ896662	Taiwan, Lieyu	[19]
	FJ896663	Taiwan, Matsu, Beigan	[19]
	FJ896664	Taiwan, Matsu, Nangan	[19]
	FJ896665	Taiwan, Matsu, Dongyin	[19]
	FJ896666	China, Zhejiang, Linan	[19]
	FJ896667	Thailand, Bangkok	[19]
	HM233928	Brazil, Paraná, Curitiba	[1]
	HM233929	Brazil, São Paulo, Ribeirão Pires	[1]
	HM233930	Brazil, Santa Catarina, Palhoça	[1]
	HM233931	China, Guangzhou, Zhongcun	[1]
	JQ712574	Argentina, Misiones, Tabay Fall	[3]
	JQ712575	Argentina, Misiones, Tabay Fall	[3]
	JQ712572	Brazil, Paraná, Foz do Iguaçu	[3]
	JQ712573	Brazil, Paraná, Foz do Iguaçu	[3]
	KX781994	Brazil, Rio Grande do Sul, Marau	[11]
	LC755005	Japan, Okinawa, Okinawa Is., Ginoza	[20]
	LC755006	Japan, Okinawa,Kumejima Is.	[20]
	LC755007	Japan, Okinawa, Ishigaki Is.	[20]
	LC755008	China, Gansu, Longnan, Bikoku	[20]
	LC755009	China, Gansu, Longnan, Bikoku	[20]
	LC755010	China, Yunnan, Yuxi Shi, Chengjiang Xian	[20]
	LC755011	China, Yunnan, Yuxi Shi, Chengjiang Xian	[20]
	PV591017	Brazil, São Paulo, Cananéia	This study (CMRP1117)
*Meghimatium rugosum* (Chen & Gao, 1982)	EF128222	Taiwan, Hualien, Zhuoxi, Neilinger Mt.	[2]
*Philomycus carolinianus (Bosc, 1802)	EF128221	USA, TN, Carter Co.	[2]

A small tissue clip was obtained from the tail end of the specimen’s foot for DNA extraction, using the QIAGEN DNEasy® Blood & Tissue Kit. The barcoding COI marker was targeted using the invertebrate primers LCO/HCO of Folmer et al. [24]. The amplification PCR protocol consisted in 3 min of initial denaturation (96°C), followed by 35 cycles of denaturation (30 s, 95°C), annealing (1 min, 48°C), and extension (2 min, 72°C), with a final 5 min extension step (72°C). The success of PCR was assessed via agarose gel electrophoresis, and the PCR product was cleaned with ExoSAP-IT™ (Affymetrix Inc.). Sanger sequencing was conducted at Macrogen Europe (Amsterdam, The Netherlands).

The sequences were *de novo* assembled and quality-checked (Phred scores) in Geneious Prime (v.2025, Biomatters Ltd.), and the consensus was uploaded to GenBank (Acc. Nr. PV591017). Further sequences of *M. pictum*, *Meghimatium* spp., and the outgroup (*Philomycus carolinianus*) were obtained from previous studies (Table 1). Sequence alignment was conducted in Geneious Prime using the MUSCLE plugin [25]. The alignment was visually proofed for inconsistencies and then subjected to a Bayesian inference phylogenetic analysis using MrBayes [v.3.2.7, 26] via the CIPRES Science Gateway [v.3.3, 27]. Two concurrent analyses with 4 Markov chains each were run for 40 million generations, discarding the first 20% as ‘burn-in’, using default priors, with nst = 6 (GTR), rates = invgamma. MCMC convergence was assessed by examining the standard deviation of split frequencies (<0.001), the potential scale reduction factor (PSRF = 1.0), and trace plots [28].

### Species distribution modelling

Species distribution modelling (SDM) was performed using MaxEnt3.4.4. [29] to evaluate potential distribution and identify possible areas of expansion of the species. Bioclimatic layers were obtained from WorldClim2.0 [30] at a spatial resolution of 4 km. To prevent multicollinearity problems, layers were submitted to Variance Inflation Factor (VIF) in a stepwise procedure, and the ones with VIF > 4 were excluded from analysis. The following variables were used in the model after this procedure: Bio 3 (Isothermality - %), Bio 7 (Temperature annual range - °C), Bio 8 (Mean temperature of wettest quarter - °C), Bio 9 (Mean temperature of driest quarter - °C), Bio 12 (Annual precipitation – mm) and Bio 14 (Precipitation of driest month – mm). To generate the model, 10,000 background points were used, with a regularisation multiplier of 1. Seventy-five per cent of presence points were used for model training and 25% for model testing, and 10 replicates were done using Bootstrap as “Replicated Run Type” [31]. Other parameters were used as default. To evaluate the model goodness of fitting, the area under the curve operating characteristics (AUC) was used. The potential distribution was computed as Cloglog. Later, with the output, the “10 percent training presence” threshold was used to define the Minimum Presence Threshold (MPT) [32]. The final output was classified as suitable (above MPT) and not suitable (below MPT) areas. To evaluate the general trend of bioclimatic variables, Partial Dependence Plots of each variable with the probability of occurrence of *M. pictum* in the final model were done.

## Results

**Fig 1:**
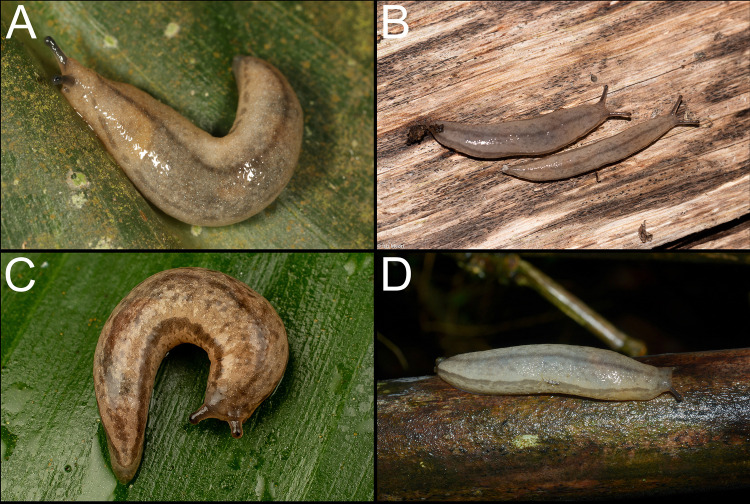
Examples of observations of *Meghimatium pictum* on iNaturalist. **A.** São Paulo, SP (https://www.inaturalist.org/observations/246793363 by Bruno Aranda, 2024; CC BY 4.0). **B.** Sapomema, PR (https://www.inaturalist.org/observations/199711105 by Ísis M. Medri, 2024; CC BY-NC 4.0). **C.** Santo André, SP (https://www.inaturalist.org/observations/170632184 by Reinaldo O. Elias, 2023; CC BY 4.0). **D.** Rio Grande, RS (https://www.inaturalist.org/observations/161678036 by Vinícius S. Domingues, 2023; CC BY 4.0).

**Fig 2:**
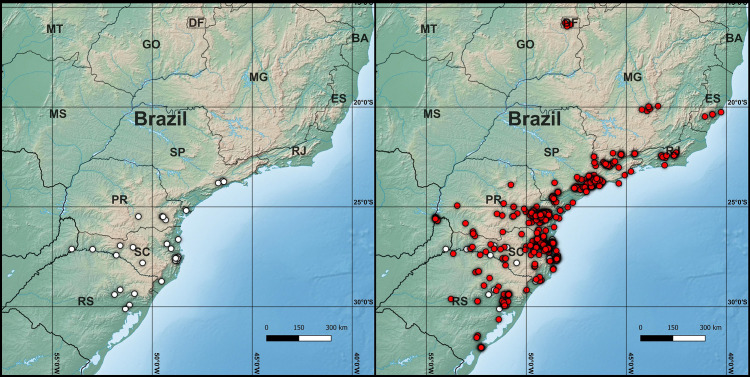
Records of *Meghimatium pictum* in Brazil up to 2011 (left) and 2024 (right). White dots represent the records previously compiled by Gomes et al. [1], while red dots indicate the new records from iNaturalist. Source of base map: Natural Earth (public domain).

As of early February 2025, our survey on iNaturalist retrieved a total of 520 observations of *Meghimatium pictum* in Brazil (Fig 1). The presence of *M. pictum* can be ascertained in the following Brazilian states: Distrito Federal (DF), Minas Gerais (MG), Espírito Santo (ES), São Paulo (SP), Rio de Janeiro (RJ), Paraná (PR), Santa Catarina (SC), and Rio Grande do Sul (RS) (Fig 2). Among these, four states (DF, MG, ES and RJ) had no previously published records of *M. pictum*.

**Fig 3:**
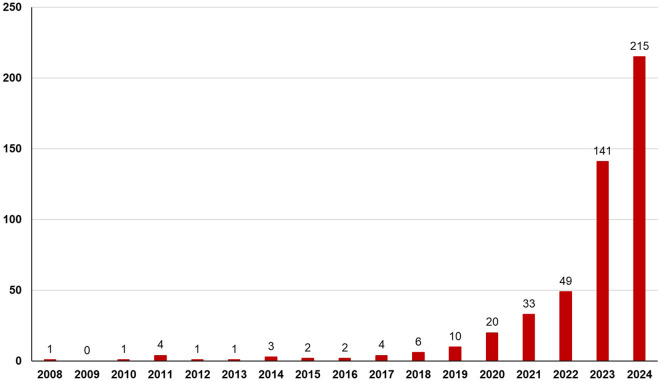
Number of records of *Meghimatium pictum* in Brazil on iNaturalist up to 2024.

The earliest record on iNaturalist dates back to July 2008 and was documented in Parque Estadual Turístico Alto do Ribeira (PETAR), a state park located in the municipality of Iporanga, São Paulo state, with a high influx of tourists. Since then, there has been a gradual increase in the number of observations per year, with a remarkable surge in 2023 and 2024 (Fig 3).

**Fig 4:**
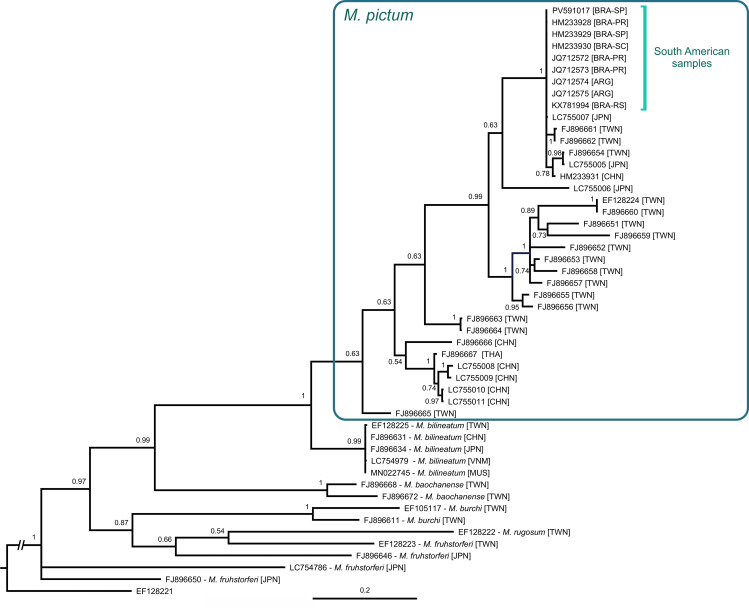
Bayesian inference phylogenetic tree (50% majority-rule consensus) based on the COI barcoding marker. Terminals show GenBank accession numbers (Table 1), including the country of origin in square brackets (in specimens from Brazil, states are also indicated: PR, Paraná; SP, São Paulo; RS, Rio Grande do Sul; SC, Santa Catarina). Posterior probabilities are shown on nodes; the scale bar represents substitutions per site.

The phylogenetic analysis used the barcoding COI marker (654 bp) and included 50 terminals, of which 35 represented *M. pictum*, with samples from Brazil, Argentina, Thailand, Japan, Taiwan and mainland China. In the resulting tree (Fig 4), the basal splits of *M. pictum* have low support (PP = 0.63), though all South American sequences were recovered in a strongly supported group (PP = 1) together with samples from Taiwan, Okinawa (Japan), and China (Guangzhou). As expected, the sequences of Brazilian and Argentinian specimens (Table 1) have 100% identity among themselves and with one of the samples from Okiwana, Japan. The identity with two samples from Taiwan and one from Guangzhou was 99%.

**Fig 5:**
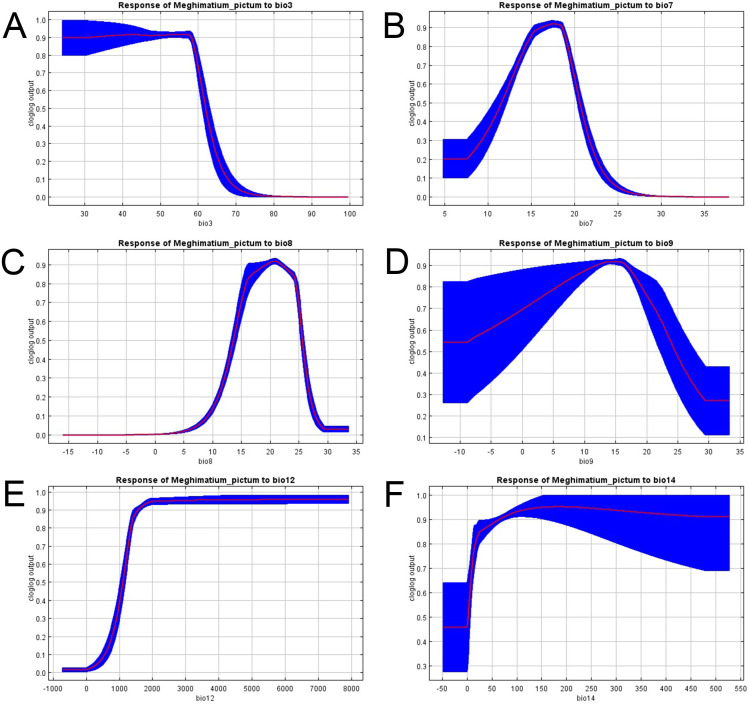
Partial dependence plots of environmental variables used in Maxent Species Distribution Modelling of *Meghimathium pictum* (Stoliczka, 1873): (A) Bio 3 (Isothermality - %), (B) Bio 7 (Temperature annual range - °C), (C) Bio 8 (Mean temperature of wettest quarter - °C), (D) Bio 9 (Mean temperature of driest quarter - °C), (E) Bio 12 (Annual precipitation – mm) and (F) Bio 14 (Precipitation of driest month – mm). Red lines indicate the probability of finding the species given each environmental variable and blue areas indicate the respective confidence intervals.

**Fig 6:**
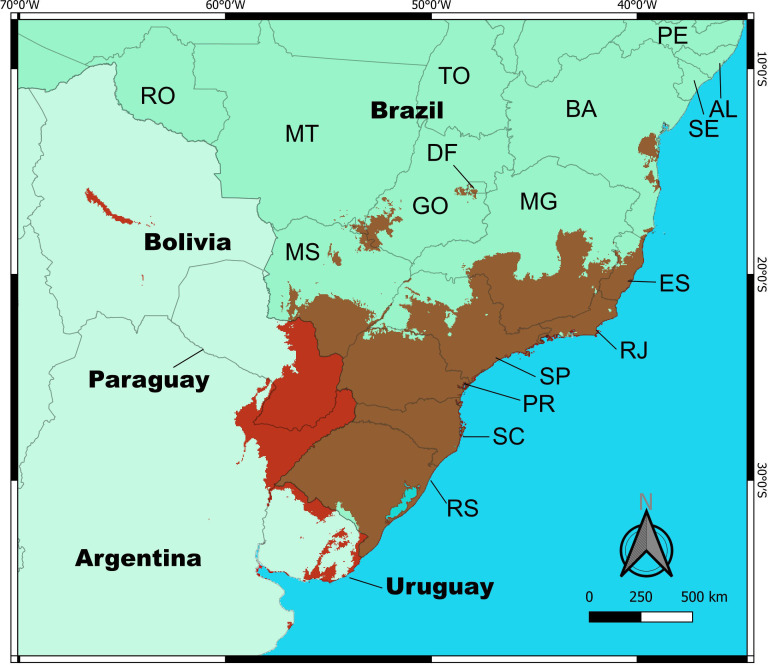
Potential distribution map of *Meghimathium pictum* (Stoliczka, 1873) in South America in thresholded format (10 percentile training presence). Legends of Brazilian States: AL = Alagoas, BA = Bahia, DF = Distrito Federal, ES = Espírito Santo, GO = Goiás, MG = Minas Gerais, MS = Mato Grosso do Sul, MT = Mato Grosso, PE = Pernambuco, PR = Paraná, RJ = Rio de Janeiro, RO = Rondônia, RS = Rio Grande do Sul, SC = Santa Catarina, SE = Sergipe, SP = São Paulo, TO = Tocantins. Source of base map: Natural Earth (public domain).

The distribution model had an AUC > 0.979 for the training and test data, with a standard deviation of 0.002, and the 10 percentile training presence threshold was 0.0285. The variable that most explained the potential habitat suitability was Bio 3 (23.4%), followed by Bio 14 (22%), Bio 9 (17.3%) and Bio 7 (15.8%) (Table 2). The other variable had less than 15% of contribution to the final results. The partial dependence plots of variables showed that Bio 12 (Fig 5E) and Bio 14 (Fig 5F) had a positive correlation with probability of occurrence, with a trend to stability after specific values (2000 and 100 mm, respectively); Bio 3 (Fig 5A) had constant values with a decrease after 60%; Bio 7 (Fig 5B, 18°C), Bio 8 (Fig 5C, 22°C) and Bio 9 (Fig 5D, 15°C) had peaks at specific values. The final generated maps showed that *M. pictum* has a large potential distribution (Fig 6). In Brazil, *M. pictum* had suitable areas in all of the southern region (Rio Grande do Sul, Santa Catarina and Paraná States), a large portion of the southeastern region (São Paulo, Minas Gerais, Rio de Janeiro and Espírito Santo States) and small parts of the midwestern (Mato Grosso do Sul, Goiás and Federal District) and northeastern (Bahia State) regions; its presence seems to be positively correlated with the Atlantic Forest ecoregion. In Argentina, it had suitable areas covering the provinces of Corrientes, Misiones, Entre-Ríos, and small parts of Buenos Aires. Additional suitable areas were also found in Uruguay and southeastern Paraguay.

**Table 2:** Percentage of contribution and general trend in partial dependence plots (positive, negative or with a peak) of environmental layers in the species distribution modeling of *Meghimathium pictum* (Stoliczka, 1873).

Variable	Contribution (%)	Trend
Bio3 – Isothermality (bio2/bio7) x 100	23.4	–
Bio 7 – Temperature annual range (bio5-bio6)	15.8	Peak
Bio 8 – Mean temperature of wettest quarter	9.8	Peak
Bio 9 – Mean temperature of driest quarter	17.3	Peak
Bio 12 – Annual precipitation	11.8	+
Bio 14 – Precipitation of driest month	22.0	+

## Discussion

The phylogenetic analysis (Fig 4) and pairwise identity of COI sequences suggest a close link between the South American populations and those from Okinawa (Japan), Taiwan, and Guangzhou (China). Considering that the area in and around Guangzhou contains the ports of Shenzhen, Guangzhou and Hong Kong (the 4th, 5th, and 8th biggest container ports in the world; [33]), it can be surmised that it is the most likely point of origin for the South American (and Okinawan) populations. The position of further specimens from mainland China and Taiwan on the COI tree (Fig 4) also hints at a complicated history of multiple introductions and potential introgression.

The numerous records on iNaturalist (520) provide an interesting insight into the invasion of *Meghimatium pictum* in Brazil, as they vastly outnumber the previous records reported roughly a decade ago by Gomes et al. [1]. In fact, there are far more observations of *M. pictum* in Brazil than in its native range in Asia on iNaturalist. This impressive number of observations, coupled with their surge in the past few years (Fig 3), is likely due to a combination of both an increase in populations of *M. pictum* in Brazil and an increase in the popularity of iNaturalist in the country (particularly compared to its small user base in Asia).

Gomes et al. [1] reported records of *M. pictum* in Brazil ranging from 1998 to 2011. From its earliest record in Curitiba, Paraná state, this species then spread to the neighboring states of São Paulo, Santa Catarina and Rio Grande do Sul [1; Fig 2]. The records reported herein show that *M. pictum* has spread further to four additional states, mostly following the distribution of the Atlantic Forest ecoregion (Fig 2). It quickly reached inland Rio de Janeiro in 2014, which was far removed from other records at the time. Records from Minas Gerais surfaced in 2017, representing the first major spread of this species away from coastal areas. In 2022, it was first reported in the Distrito Federal, near the Brazilian capital Brasília in the center of the country, and two additional records in the area have appeared in the following years.

*Meghimatium pictum* is a highly versatile species found in a wide range of habitats in Brazil, ranging from undisturbed forests to urban areas [1]. Worryingly, it has already been recorded in several protection areas across its current distribution [1; herein]. So far, most of its occurrence range in Brazil is restricted to southern and southeastern Atlantic Forest areas (Fig 2), but its recent introduction to the Distrito Federal, within the Cerrado ecoregion, suggests that it can also spread to different ecoregions and drier climates.

Our species distribution model supports the notion that suitable areas for *M. pictum* are mostly restricted to subtropical Brazil and northeastern Argentina, largely correlated with areas originally covered by the Atlantic Forest ecoregion. The Atlantic Forest is a large tropical rainforest that once spread across most of the eastern coast of Brazil, although only 7% of its original natural cover remains today, and is considered a biodiversity hotspot due to its large number of endemic species and long history of exploitation [34]. Climatic and environmental factors related to this ecoregion (e.g., dense vegetation, relatively stable temperature, high humidity) may influence the suitability of these areas for *M. pictum*. Additionally, most of the large urban areas in Brazil are located in areas that formerly belonged to the Atlantic Forest, which could also aid in the spread of a synanthropic exotic species such as *M. pictum*.

While most of the modelled suitable range in Brazil is contiguous, a few isolated areas in the southwestern border of Goiás state and the coastal region of Bahia state were also found to be suitable for *M. pictum* (Fig 6). Remarkably, there are currently no records of *M. pictum* in those areas, but future surveys should keep an eye out for this species’ arrival or still-unrecorded presence.

Although the large number of records of *M. pictum* helped us to better model its distribution in Brazil, sampling bias should also be taken into consideration when interpreting these data. As discussed by previous studies, records from iNaturalist are not a perfect representation of a species’ occurrence, since they are also affected by several biases related to the observers and tend to cluster around areas with large human populations [17]. As such, the lack of records of *M. pictum* in isolated areas may simply be due to lack of sampling effort in those areas and not an indication of absence.

Invasive slugs often pose significant challenges to the areas they colonize. Since its introduction in Brazil, *M. pictum* has been considered an agricultural pest in vineyards and strawberry crops, leading to economic losses by damaging the fruits, impairing grape sales, and lowering the quality of wine and juice [9,35]. Those publications labelled *M. pictum* as an agricultural pest, though the evidence presented in them is anecdotal, lacking a measurement and assessment of actual impacts. Thus, no thorough study of the impacts of *M. pictum* has been conducted yet in South America, particularly in comparison to the impacts of other common non-native slugs that are problematic to crops (e.g., *Deroceras* spp.). Such data is necessary to decide which actions (if any) should be taken in relation to *M. pictum*.

*Meghimatium pictum* has also been identified as an intermediate host for *Angiostrongylus costaricensis*, a nematode responsible for abdominal angiostrongyliasis in humans. Notably, *M. pictum* has already been linked to human infection following the diagnosis of abdominal angiostrongyliasis in a grape farmer in southern Brazil, likely resulting from the consumption of grapes from slug-infested plants [11,12]. Even though this remains an isolated case, given the rapid spread of *M. pictum* across the country, it is expected that more such cases will occur; thus, it will be worthwhile for public health officials to keep track of them and, when necessary, inform people in areas of potential exposure. The “good old” prophylaxis for a variety of parasites in Brazil also apply in this case, i.e., washing vegetables prior to eating to remove slugs and their slime. Still, farmers exposed to slug-infested crops could benefit from extra protection (e.g., wearing gloves during work), as the infective L3 larvae of *A. costaricensis* can reportedly remain infective for up to 17 days [11].

Control measures against *M. pictum*, if even feasible at this stage of its invasion, are still poorly understood. Pesticides, such as iron phosphate baits, have proven effective against *M. pictum* [10,36], but further research to explore alternative strategies is urgently needed, as these types of pesticides have deleterious effects in animals that feed on slugs, such as birds, with increasing severity in the “upper strata” of the food chain through bioaccumulation [e.g., 37]. Since the actual extent of its impacts on the local ecosystems, crops, and human health are also poorly understood, additional monitoring should be undertaken to determine whether control measures are necessary.

## Conclusion

Despite the fast spread of this species in Brazil and the potential problems it raises, research on *M. pictum* remains scarce in the country and the species is rarely addressed in discussions on invasive species [e.g., 38]. Our results suggest that Guangzhou, mainland China, is the most likely point of origin for the South American populations of *M. pictum*, as well as the closely related populations from Okinawa, shedding some light on the history of its spread outside its native range. Coupled with the newly compiled records, this allows us to better understand how *M. pictum* reached Brazil and how it is spreading across the country over time since its introduction.

Although biological invasions pose a serious risk of negative environmental and socioeconomic impacts, there are still large knowledge gaps concerning the exotic mollusks introduced in South America [39]. As such, efforts to understand these introduced species are extremely important, particularly in defining whether they are indeed invasive. This updated assessment on the status of *M. pictum* should therefore serve as an example, stressing the need for increased monitoring and an evaluation of actual impacts (if any) to decide on whether control measures are necessary. As the mantleslug continues to expand its range in Brazil (and South America), its movements should be closely monitored in the coming years.

As evidenced by previous studies, iNaturalist and similar citizen science platforms are valuable tools for monitoring biological invasions, from terrestrial gastropods [18,40,41] to other invertebrates [e.g., 42] and plants [e.g., 43]. Their main advantage lies in the ability to generate large amounts of data across extensive areas within a short timeframe, enabling more rapid tracking of invasions compared to traditional surveys. While this data is mostly useful for documenting species distributions, it is insufficient for assessing their abundance due to several observer-related biases [17]. Therefore, citizen science data should increasingly be incorporated into future studies to complement traditional surveys and provide a better understanding of species distributions.

## Supporting information

S1 File Supplementary material. (CSV)
